# Expression of SASP, DNA Damage Response, and Cell Proliferation Factors in Early Gastric Neoplastic Lesions: Correlations and Clinical Significance

**DOI:** 10.3389/pore.2022.1610401

**Published:** 2022-08-19

**Authors:** Li Liang, Yijie Chai, Fei Chai, Haijing Liu, Ningning Ma, Hong Zhang, Shuang Zhang, Lin Nong, Ting Li, Bo Zhang

**Affiliations:** ^1^ Department of Pathology, Peking University First Hospital, Beijing, China; ^2^ Department of Pathology, School of Basic Medical Sciences, Peking University Health Science Center, Beijing, China; ^3^ Department of Pathology, Fenyang College of Shanxi Medical University, Fenyang, China

**Keywords:** immunohistochemistry, gastric cancer, p53, DNA damage response, endoscopic submucosal dissection, senescence-associated secretory phenotype

## Abstract

The cyclic GMP-AMP synthase (cGAS)-stimulator of interferon genes (STING)-mediated senescence-associated secretory phenotype (SASP) pathway has recently been identified in the suppression and promotion of cancers. However, its practical role in carcinogenesis remains to be comprehensively elucidated. Here, we describe an investigation analysing SASP activity and its correlations with DNA damage response (DDR), genomic mutations, and cell proliferation in gastric carcinogenesis among 30 cases with available endoscopic submucosal dissection (ESD) specimens of early neoplastic lesions (including low-grade dysplasia [LGD], high-grade dysplasia [HGD], and intramucosal carcinoma). The positive cells of senescence-associated β-galactosidase staining and cGAS, STING, interferon-regulatory factor 3 (IRF3), and signal transducer and activator of transcription 6 (STAT6) expression levels using immunostaining were elevated in HGD and in cancers. Similarly, increased expression of the Fanconi anemia group D2 (FANCD2) protein, tumour suppressor p53 binding protein 1 (TP53BP1), and replication protein A (RPA2) (i.e., primary DDR factors) was detected in HGD and in cancers; these increased expression levels were closely correlated with high expression of Ki67 and minichromosome maintenance complex component 7 (MCM7) proteins. Moreover, genomic mutations in *TP53* gene were detected in 56.67% of the evaluated cases (17/30) using next-generation sequencing, and positive staining was verified in HGD and in cancers. Statistical analysis revealed that cell proliferation closely correlated with the expression of DDR factors, of which TP53BP1 was positively associated with SASP factors and IRF3 was positively correlated with cell proliferation. In addition, an analysis evaluating clinical features demonstrated that STAT6-positive cases showed a longer progression-free survival time than STAT6-negative cases. Our evaluation, conducted using a limited number of specimens, suggests SASP may be prevalent in early gastric neoplastic lesions and could be activated by accelerated cell proliferation-induced DDR. The clinical significance of SASP still needs to be determined.

## Introduction

Senescent cells can produce pro-inflammatory factors, including cytokines, growth factors, proteases, and chemokines; this state is collectively termed the senescence-associated secretory phenotype (SASP) [1, 2]. Recently, SASP has been shown to be initiated by cyclic GMP-AMP synthase (cGAS) and the cyclic GMP-AMP synthase (cGAS)-stimulator of interferon genes (STING) pathway. Once bound to cytosol-free DNA, cGAS catalyses the formation of cyclic guanosine monophosphate–adenosine monophosphate (2ʹ,3′-cGAMP), a secondary messenger, thereby activating STING. This then activates downstream signalling of TANK-binding kinase 1 (TBK1), interferon-regulatory factor 3 (IRF3), signal transducer and activator of transcription 6 (STAT6), nuclear factor kappa B, and CCAAT enhancer-binding protein beta (C/EBPbeta). The amplified signalling cascade stimulates the production of pro-inflammatory factors, such as type I interferon [1, 3, 4]. As innate immune defensive machinery, SASP is crucial to eradicating foreign pathogens [5]. The activation of SASP is triggered by the binding of cGAS to DNA. Cellular DNA (usually released in DNA damage) could likewise stimulate SASP activity in order to clear senescent or damaged cells [6, 7]. SASP has also been identified as being involved in various pathogenic conditions and states, including inflammation, autoimmune disease, and tumorigenesis. Moreover, SASP-activated senescent cells exhibit tumour suppressive functions, thereby preventing the growth of cancer cells, and induce cancer cell genomic instability and lead to the remodelling of the tumour microenvironment in either an autocrine or paracrine manner [8, 9]. Thus far, although SASP activity in cancers has been documented in advanced cancers, research on its role in early cancer development and progression remains limited.

Gastric cancers are widely distributed throughout the world, and are especially prevalent in East Asian countries [10]. Although the mechanisms underlying gastric carcinogenesis have been extensively explored, gastric cancer progression has not been thoroughly investigated [11, 12]. Recently, genetic research has shown that *TP53* mutations and genetic instability are generally involved in the pathogenesis of this gastric cancer; however, the underlying mechanisms remain to be elucidated [13–15]. Nevertheless, the step-wise development of gastric cancers (i.e., from abnormal proliferation, to dysplasia, and subsequently to cancer transformation) has been well defined. With the early detection of neoplastic lesions using coupled endomicroscopy and biopsy, endoscopic submucosal dissection (ESD) and endoscopic mucosal resection have been introduced into the early treatment of gastric cancers, thereby meaningfully improving clinical outcomes [16–18]. ESD is also a suitable method for the analysis of gastric carcinogenesis.

In the present investigation, ESD specimens from patients with early gastric cancer and precancerous lesions were evaluated. SASP factors, DNA damage response (DDR), genetic mutations, and cell proliferation were analysed to determine the role of SASP as well as its correlations with DDR, *TP53* mutations, and cell proliferation.

## Materials and Methods

### Patients and ESD Samples

Thirty ESD specimens from patients with early gastric cancer were selected from all ESD specimens available at the Department of Pathology (Peking University First Hospital) from 2016 to 2018. Clinical information for these specimens was abstracted from patient medical records. In addition, five fresh ESD specimens were collected. Pathological evaluation was conducted based on the 5th World Health Organization classification system [18]. The distribution of neoplastic lesions included 23 cases of low-grade dysplasia (LGD), 24 cases of high-grade dysplasia (HGD), 30 cases of intramucosal carcinoma, and 11 cases of submucosally invasive carcinoma (pT1b1, 7/30; pT1b2, 4/30). A total of 19 cases of well-differentiated gastric adenocarcinoma and 11 cases of poorly differentiated gastric adenocarcinoma were evaluated (Table 1).

**TABLE 1:** The distribution of lesions in 30 cases of ESD specimens.

Case NO.	LGD	HGD	Cancer	Differentiation	Stage
1		√	√	Well	pT1b1
2	√	√	√	Well	pT1a
3	√	√	√	Well	pT1b1
4	√		√	Poor	pT1b2
5	√	√	√	Poor	pT1a
6			√	Poor	pT1b1
7	√	√	√	Well	pT1a
8	√	√	√	Well	pT1b1
9	√		√	Well	pT1a
10	√	√	√	Well	pT1b2
11			√	Poor	pT1b1
12	√	√	√	Well	pT1a
13	√	√	√	Well	pT1a
14	√	√	√	Well	pT1a
15	√		√	Well	pT1a
16	√	√	√	Well	pT1a
17	√	√	√	Well	pT1b2
18	√	√	√	Poor	pT1a
19	√	√	√	Well	pT1a
20	√	√	√	Poor	pT1b1
21	√	√	√	Poor	pT1b1
22	√	√	√	Well	pT1b2
23	√	√	√	poor	pT1a
24	√	√	√	Well	pT1a
25	√	√	√	Well	pT1a
26		√	√	Well	pT1a
27		√	√	Well	pT1a
28		√	√	Poor	pT1a
29	√		√	Poor	pT1a
30		√	√	Poor	pT1a

ESD, endoscopic submucosal dissection; HGD, high-grade dysplasia; LGD, low-grade dysplasia.

The 30 included patients comprised 24 male and 6 female patients, with ages ranging from 43 to 79 years (median, 65 years). A total of 12 cases involved the pyloric antrum, whereas the other 18 cases involved the body or fundus. Clinical information and follow-up data were obtained for all cases. The follow-up period was defined as starting from the date of initial diagnosis and ending at the date of the patient’s death, progression, relapse, or last follow-up visit. The follow-up duration ranged from 10 to 54 months (median, 36.5 months). Frozen sections from five additional ESD specimens derived from patients with early gastric carcinomas were subjected to SA-β-gal staining.

### Senescence-Associated β-galactosidase Staining

Frozen sections from fresh ESD specimens were fixed in 4% formalin and evaluated using an SA-β-gal staining kit (GenMed Scientifics, Inc., Wilmington, DE, United States) according to the manufacturer’s instructions. Briefly, tissues were stained with the SA-β-gal staining solution overnight at 37°C; we counted 100 cells in random fields, and calculated the percentage of SA-β-gal-positive cells (i.e., blue cells).

### Immunohistochemistry and Evaluation

Formalin-fixed, paraffin-embedded (FFPE) sections were deparaffinized with serial xylene treatments and hydrated in graded alcohols. Endogenous peroxidase activity was quenched using 0.3% hydrogen peroxide for 60 min. Antigen retrieval was carried out by heating the specimen in citrate buffer (20-mM citrate buffer, pH 6.0) at 95°C for 20 min. After blocking with horse serum (1:100 in phosphate buffered saline, PBS), the sections were incubated with primary antibodies in various dilutions overnight at 4°C. The specimens were incubated using a Dako Envision Flex amplification kit (Dako, Glostrup, Denmark) for 60 min, and colour development was completed using a freshly prepared diaminobenzidine solution (Dako). The sections were then counterstained using Mayer’s haematoxylin. For the negative control, the primary antibody was replaced with phosphate buffered saline (PBS) or normal rabbit serum. The relevant antibodies and their associated information are summarized in Supplementary Table S1.

We found that cGAS, STING, and IRF3 demonstrated cytoplasmic staining, while STAT6, Ki67, minichromosome maintenance complex component 7 (MCM7), and p53 showed nuclear staining. The evaluation was performed in whole slides. Since the intensive staining for each of these factors was similar, the positive stained glandular epithelial cells were semiquantitatively estimated and the resultant percentages represent the positive glandular epithelial cells against total glandular cells for each of groups. Stromal cells were not counted. For associations with clinicopathological parameters and Kaplan–Meier single-factor analysis, positive cases were defined as those with either >10% or >20% positive tumour cells (i.e., different thresholds were evaluated), while negative cases were defined as those with ≤10% or ≤20% positive tumour cells.

### Next-Generation Sequencing

DNA was extracted from FFPE blocks derived from 30 cases using a QIAamp DNA FFPE tissue kit (Qiagen, Hilden, Germany). The DNA library was constructed using the capture method, and paired-end sequencing was performed using a NextSeq 500 Sequencer in combination with the NextSeq™ 500 High Output Kit (Illumina, Inc., San Diego, CA, United States) according to the manufacturer-recommended protocols for gastrointestinal tumour-related genes (Burning Rock, Guangzhou, China). The average sequencing depth was 1,000× for tissue samples. Single nucleotide variants, copy number variants, and fusion were called in the pipeline. Target regions were captured using designed probes spanning 41 genes (Supplementary Table S2).

Sequence data were mapped onto the reference human genome (hg19) using Burrows–Wheeler Aligner version 0.7.10. Local alignment optimization, duplication marking, and variant calling were performed using the Genome Analysis Tool Kit version 3.2 and VarScan version 2.4.3.

### Statistical Analysis

All statistical analyses were performed using SPSS statistical software (version 17.0, Chicago, IL, United States), Microsoft Excel 2007 (Seattle, WA, United States), and GraphPad Prism software (San Diego, CA, United States). The data obtained from immunohistochemistry (IHC) and association between expressed factors and other parameters were analysed using chi-square tests, Fisher exact tests, Mann–Whitney U tests, and Spearman correlation analysis. The distributions of IHC results between different lesion types were analysed using nonparametric Friedman tests. Progression-free survival curves were plotted using the Kaplan-Meier method and were compared using log-rank tests. Differences were considered statistically significant given a two-sided *p* value of <0.05.

## Results

### Expression of SASP Factors in Early Gastric Neoplastic Lesions

**FIGURE 1:**
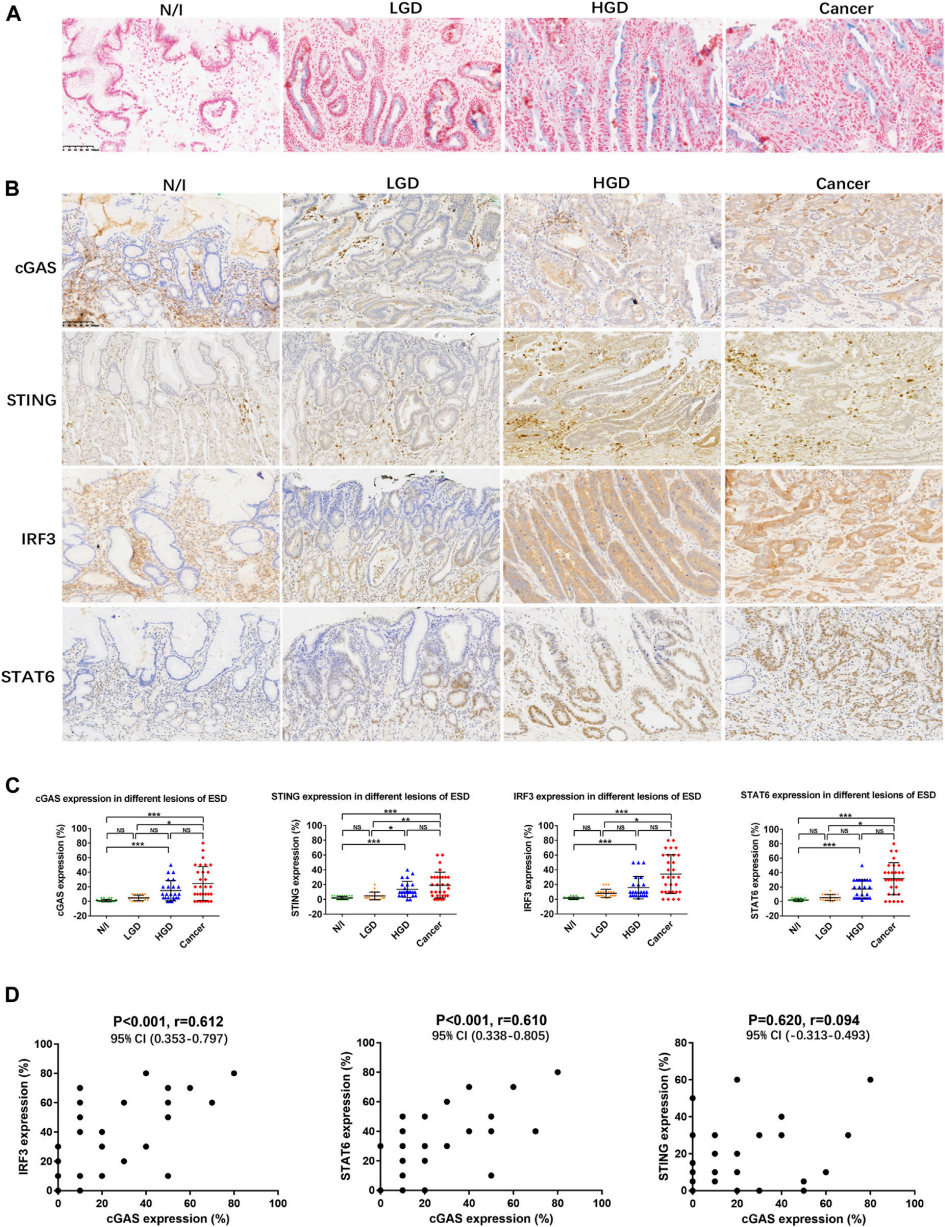
SASP activity in gastric lesions. **(A)** SA-β-gal staining in frozen ESD specimens. No positive SA-β-gal staining was detected in normal or inflammatory gastric mucosa. Scattered positive staining (shown as blue cells) was detected in the LGD cytoplasm. More positive cells were found in HGD and in early gastric cancer. **(B)** Expression of SASP factors, assessed using immunohistochemistry. **(C)** Distribution of the expression of SASP factors in different ESD lesions. The positive expression of cGAS, STING, IRF3, and STAT6 in each of the lesions was calculated using percentages; the distributions are displayed herein. The distribution of positive expression between different lesions was analysed using the Friedman test. Positive staining for cGAS, STING, IRF3, and STAT6 in carcinomas was statistically significantly different from that in either N/I or LGD. However, there were no differences in findings between HGD and carcinomas. **(D)** Mutual correlations were analysed using Spearman correlation based on immunohistochemistry results for cGAS, STING, IRF3, and STAT6 (A, B; magnification, ×200). Abbreviations: cGAS, cyclic GMP-AMP synthase; ESD, endoscopic submucosal dissection; HGD, high-grade dysplasia; IRF3, interferon-regulatory factor 3; LGD, low-grade dysplasia; N/I, normal or inflammatory; NS, no statistical significance; SA-β-gal, senescence-associated β-galactosidase; SASP, cyclic GMP-AMP synthase (cGAS)-stimulator of interferon genes (STING)-mediated senescence-associated secretory phenotype; STAT6, signal transducer and activator of transcription 6; STING, cyclic GMP-AMP synthase (cGAS)-stimulator of interferon genes. The level of statistical significance was indicated as follows: * < 0.05, ** < 0.01, and *** < 0.001.

Fresh frozen sections from five ESD specimens of early gastric carcinomas were subjected to SA-β-gal staining. Results showed positive staining in the cytoplasm of precancerous and cancerous cells in all evaluated cases (>10% SA-β-gal-positive cells) but not in the inflammatory gastric mucosa (Figure 1A).

Expression of cGAS, STING, IRF3, and STAT6 factors involved in the mediation of SASP activity was analysed in the 30 ESD specimens. cGAS staining was mainly detected in the cytoplasm of precancerous and cancerous cells (Figure 1B), while STING staining was detected in the cytoplasm of cancer cells and stromal cells such as lymphocytes (Figure 1B). In addition, the positive expression of IRF3 presented as cytoplasmic staining in precancerous and cancerous lesions, while the positive expression of STAT6 mainly presented as nuclear staining in precancerous and cancerous lesions (Figure 1B). The distributions of cGAS, STING, IRF3, and STAT6-positive expression was similar in high-grade dysplastic and cancerous lesions, with diffuse positive staining. Focal or scattered weak staining was detected in low-grade dysplastic cells (Figure 1B). The distribution of positive expression between different lesions was analysed using non-parametric Friedman tests. Positive staining for cGAS, STING, IRF3, and STAT6 in the evaluated carcinomas was statistically significantly different from that detected in normal or inflammatory (N/I) cells or in LGD; there were no differences in expression between HGD and the evaluated carcinomas (Figure 1C).

On performing Spearman correlation, we found that cGAS expression positively correlated with IRF3 and STAT6 expression, but not with STING expression (Figure 1D; Table 2). We also found significantly positive correlation between IRF3 and STAT6 expression, but not between STING and IRF3 or STAT6 expression (Table 2). These results suggest that SASP may be prevalently activated in early gastric carcinogenesis.

**TABLE 2:** Correlations between SASP, DDR, and proliferation factors in early gastric cancer.

	STING	IRF3	STAT6	FANCD2	TP53BP1	RPA2	Ki67	MCM7
cGAS	*p* = 0.620	*p* < 0.001*	*p* < 0.001*	*p* = 0.324	*p* = 0.014*	*p* = 0.236	*p* = 0.188	*p* = 0.165
	*r* = 0.094	*r* = 0.612	*r* = 0.610	*r* = 0.186	*r* = 0.443	*r* = 0.223	*r* = 0.247	*r* = 0.260
STING		*p* = 0.274	*p* = 0.246	*p* = 0.141	*p* = 0.039*	*p* = 0.246	*p* = 0.253	*p* = 0.275
		*r* = 0.206	*r* = 0.219	*r* = 0.275	*r* = 0.380	*r* = 0.219	*r* = 0.216	*r* = 0.206
IRF3			*p* = 0.005*	*p* = 0.008*	*p* < 0.001*	*p* = 0.009*	*p* = 0.003*	*p* = 0.002*
			*r* = 0.504	*r* = 0.477	*r* = 0.625	*r* = 0.470	*r* = 0.520	*r* = 0.537
STAT6				*p* = 0.025*	*p* = 0.114	*p* = 0.224	*p* = 0.724	*p* = 0.947
				*r* = 0.410	*r* = 0.295	*r* = 0.229	*r* = 0.067	*r* = 0.013
FANCD2					*p* < 0.001*	*p* = 0.013*	*p* = 0.024*	*p* = 0.060
					*r* = 0.612	*r* = 0.450	*r* = 0.412	*r* = 0.348
TP53BP1						*p* = 0.023*	*p* = 0.010*	*p* = 0.011*
						*r* = 0.413	*r* = 0.462	*r* = 0.460
RPA2							*p* = 0.041*	*p* = 0.011*
							*r* = 0.375	*r* = 0.458
Ki67								*p* < 0.001*
								*r* = 0.697

cGAS, cyclic GMP-AMP synthase; FANCD2, Fanconi anemia group D2; IRF3, interferon-regulatory factor 3; MCM7, minichromosome maintenance complex component 7; RPA2, replication protein A; STAT6, signal transducer and activator of transcription 6; STING, cyclic GMP-AMP synthase (cGAS)-stimulator of interferon genes; TP53BP1, tumour suppressor p53 binding protein 1.

*Indicates statistical significance. Statistical analyses were carried out using Spearman correlation.

### Expression of DDR Factors in Early Gastric Neoplastic Lesions

**FIGURE 2:**
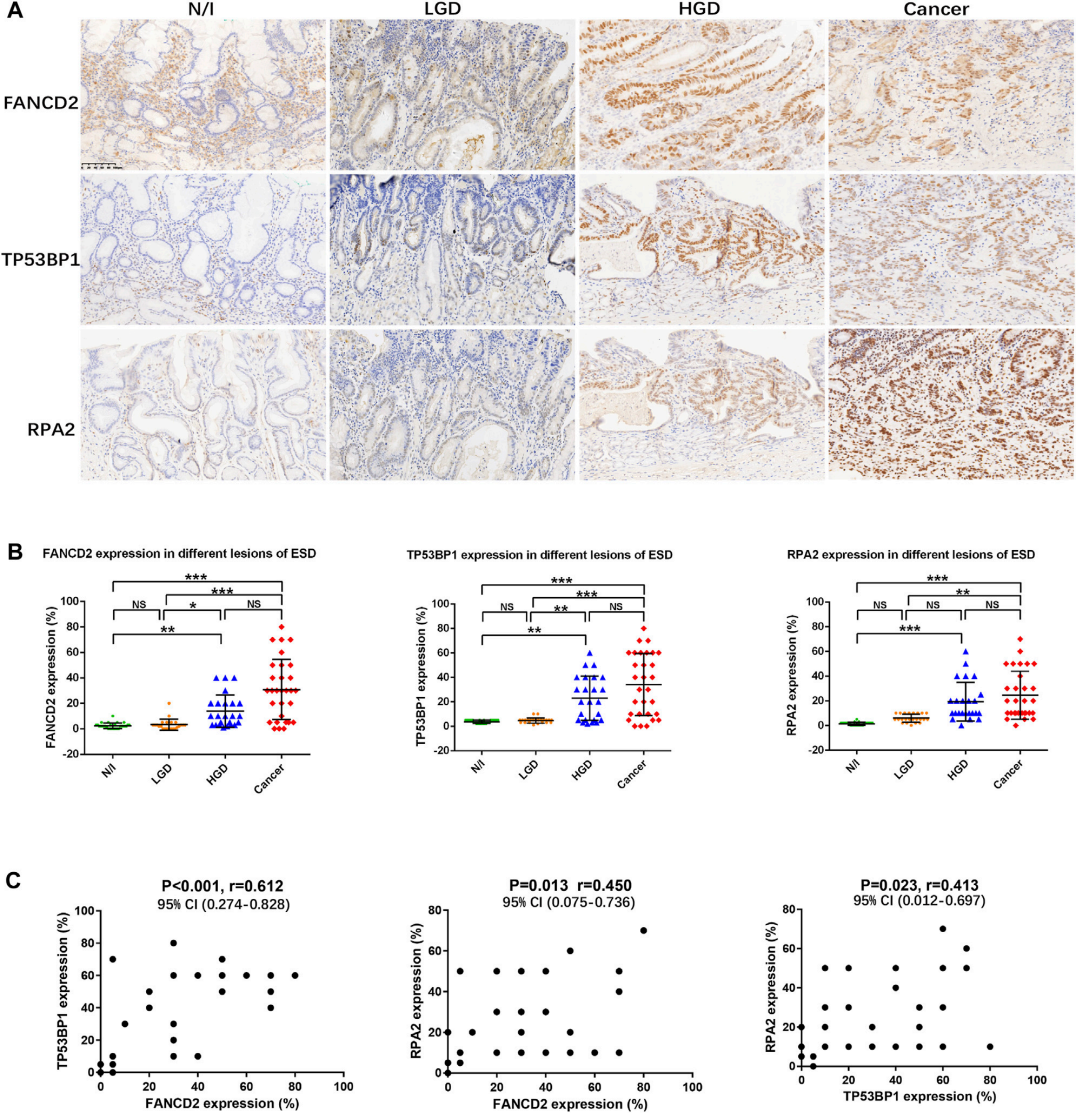
Expression of DDR in gastric lesions. **(A)** Expression of DDR factors (FANCD2, TP53BP1, and RPA2), as assessed using immunohistochemistry. **(B)** Distribution of the expression of DDR factors in different lesions using ESD. The positive expression of FANCD2, TP53BP1, and RPA2 in each of the lesions was calculated using percentages; their distributions are displayed herein. The results of Friedman tests demonstrated that the expression levels of FANCD2, TP53BP1, and RPA2 were elevated in HGD and in carcinomas, as compared with in N/I or LGD. **(C)** Mutual correlations were analysed using Spearman correlation based on immunohistochemistry results for FANCD2, TP53BP1, and RPA2 **(A)** (magnification, ×200). Abbreviations: DDR, DNA damage response; ESD, endoscopic submucosal dissection; FANCD2, Fanconi anemia group D2; LGD, low-grade dysplasia; HGD, high-grade dysplasia; N/I, normal or inflammatory; NS, no statistical significance; RPA2, replication protein A; TP53BP1, tumour suppressor p53 binding protein 1. The level of statistical significance was indicated as follows: * < 0.05, ** < 0.01, and *** < 0.001.

SASP activation is induced by the binding of cGAS to free cytoplasmic DNA generated following DNA damage [3, 4]. To determine DNA damage in early gastric cancer, factors involved in DDR were estimated using IHC. The expression of the Fanconi anemia group D2 (FANCD2) protein, tumour suppressor p53 binding protein 1 (TP53BP1), and replication protein A (RPA2) indicated nuclear staining to varying degrees in precancerous and cancerous lesions (Figure 2A). Expression levels of FANCD2, TP53BP1, and RPA2 in each lesion (i.e., from every case) were calculated; their distributions are shown in Figure 2B. The expression levels of FANCD2, TP53BP1, and RPA2 were statistically significantly elevated in HGD and in carcinomas, according to the results of non-parametric Friedman tests (Figure 2B).

Mutual correlations were also statistically evaluated using Spearman correlation tests, the results of which indicated statistically significantly positive correlations between FANCD2, TP53BP1, and RPA2 expression, with a closer correlation detected between FANCD2 and TP53BP1 expression (Figure 2C; Table 2).

### Genomic Mutations in Early Gastric Neoplastic Lesions

**FIGURE 3:**
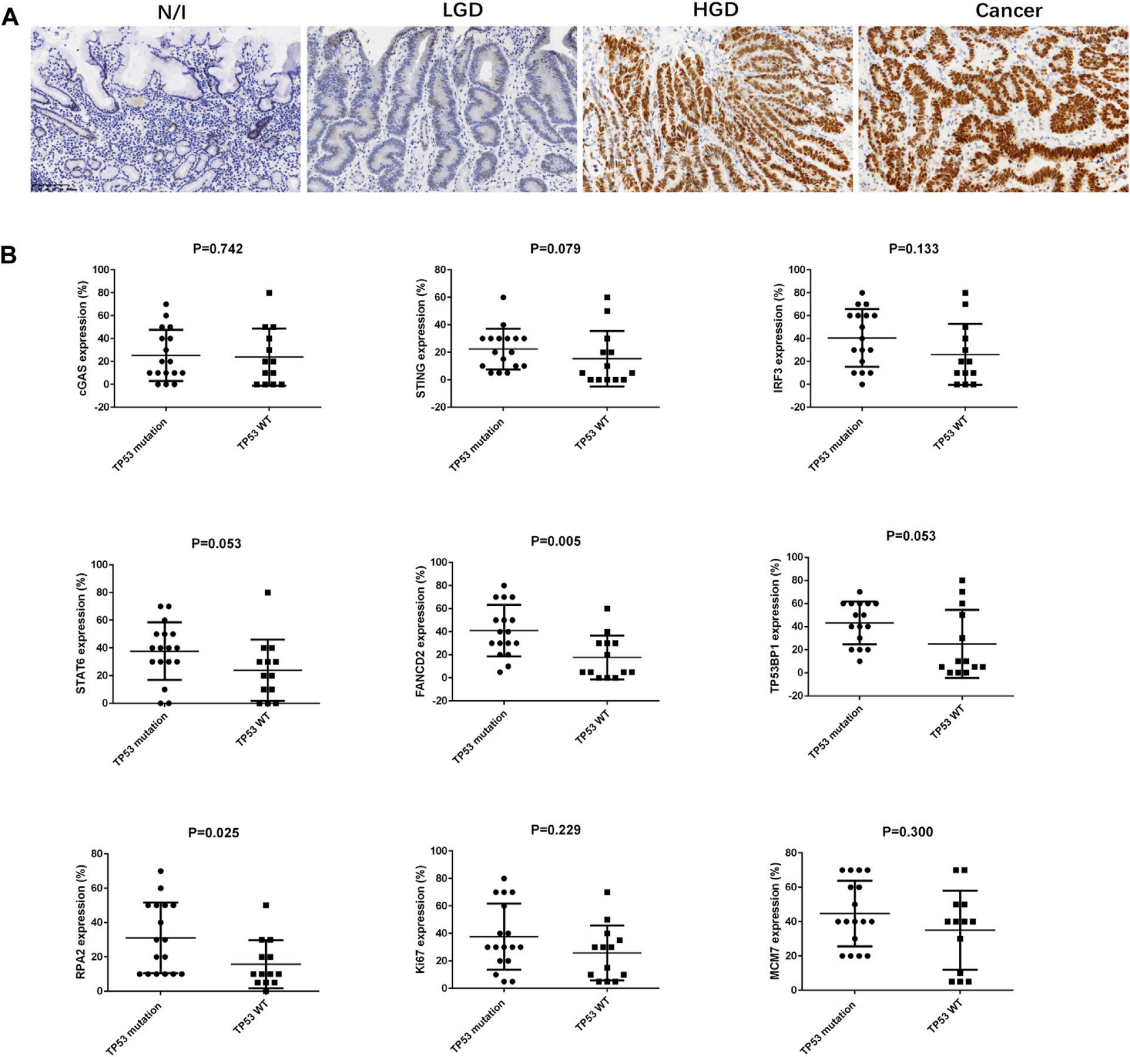
Immunostaining for p53 in gastric lesions and the association between TP53 mutation and SASP, DDR, and proliferation factors in early gastric cancer. **(A)** Expression of p53, as assessed using immunohistochemistry. **(B)** The association between TP53 mutation and SASP, DDR, and proliferation factors in early gastric cancer were analysed using Mann-Whitney U-test. Abbreviations: HGD, high-grade dysplasia; LGD, low-grade dysplasia; N/I, normal or inflammatory; SASP, cyclic GMP-AMP synthase (cGAS)-stimulator of interferon genes (STING)-mediated senescence-associated secretory phenotype; c GAS, cyclic GMP-AMP synthase; STING, cyclic GMP-AMP synthase (cGAS)-stimulator of interferon genes; IRF3, interferon-regulatory factor 3; STAT6, signal transducer and activator of transcription 6; DDR, DNA damage; FANCD2, Fanconi anemia group D2; TP53BP1, tumour suppressor p53 binding protein 1; RPA2, replication protein A.

All cases were subjected to targeted sequencing using a panel of 41 genes expressed in gastric cancers. The detected genomic mutations are summarized in Supplementary Table S3. In addition to the scattered distribution of breast cancer genes (*BRCA*), the ataxia telangiectasia mutated gene (*ATM*), adenomatous polyposis coli (*APC*), phosphatase and tensin homolog (*PTEN*), and Kirsten rat sarcoma viral oncogene *(KRAS*) mutations, our results showed that 17 cases (56.67%) harboured *TP53* gene mutations in the coding sequence hot spots from exon 4 to exon 9 (Table 3). Moreover, 20 cases were also stained for immunohistochemical analysis. In the evaluation of p53 expression, we found that cases with genomic mutations always presented with strongly positive staining (i.e., in ≥90% of cells) for HGD and for carcinomas, while cases without mutations exhibited weak and scattered positive cells (similar to the findings described in a recent report) [19]. All nine cases with missense mutation were positive on IHC. In addition, one of four cases with frameshift mutations was positive, while one case with a splice-site mutation was negative on IHC. Seven cases with wild-type sequencing were negative. Positive staining for p53 was only present in HGD and in cancer cells; these specimens showed approximately 90% positive cells (Figure 3A; Table 3).

**TABLE 3:** *TP53* gene mutations and IHC findings in 30 cases with available ESD specimens.

Case	IHC (%*)	Mutation	Mutation location
1	ND	Splicing	Exon 5, c.376-2A>G
2	ND	Nonsense	Exon 4, c.159G>A
3	Positive (95%)	Missense	Exon 6, c.641A>G
4	Positive (90%)	Missense	Exon 8, c.818G>A
5	Positive (95%)	Missense	Exon 7, c.733G>A
6	Positive (95%)	Frameshift	Exon 9, c.956del
7	Positive (95%)	Missense	Exon 8, c.844C>T
8	ND	Nonsense	Exon 8, c.915C>T
9	ND	WT	WT
10	Positive (95%)	Missense	Exon 7, c.733G>A
11	ND	WT	WT
12	ND	Frameshift	Exon 5, c.491_428del
13	Negative	WT	WT
14	ND	WT	WT
15	ND	WT	WT
16	Negative	Splicing	Intron 9, c.994-1G>A
17	Positive (95%)	Missense	Exon 8, c.817C>T
18	ND	WT	WT
19	Positive (90%)	Missense	Exon 8, c.844C>T
20	Negative	WT	WT
21	Negative	Frameshift	Exon 5, c.463_466del
22	Positive (90%)	Missense	Exon 8, c.818G>A
23	Positive (90%)	Missense	Exon 8, c.817C>T
24	Negative	WT	WT
25	Negative	Frameshift	Exon 7, c.675-5_675dup
26	Negative	WT	WT
27	Negative	WT	WT
28	Negative	WT	WT
29	Negative	WT	WT
30	ND	WT	WT

ESD, endoscopic submucosal dissection; HGD, high-grade dysplasia; IHC, immunohistochemistry; ND, IHC not performed; TP53, tumour protein 53; WT, wild-type.

*Percentage of positive cells in HGD and in cancer specimens.

### Ki67 and MCM7 Expression in Early Gastric Neoplastic Lesions

**FIGURE 4:**
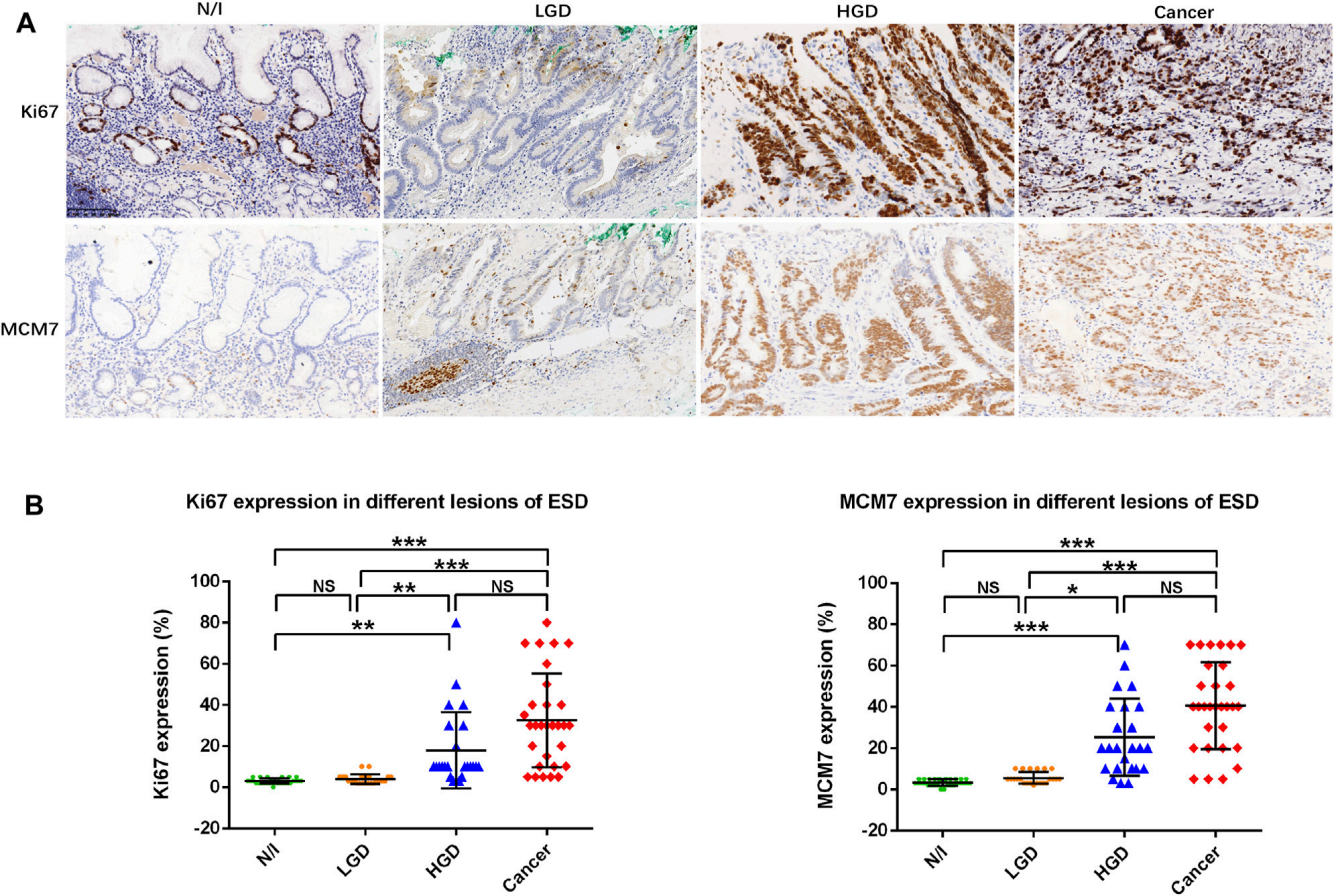
Ki67 and MCM7 expression in gastric lesions. **(A)** Positive staining for Ki67 and MCM7 was mainly localized to the proliferation zone of normal or inflammatory gastric mucosa. Expression patterns showed scattered nuclear staining in LGD, whereas positive staining was markedly stronger in HGD and in gastric cancer cells. **(B)** Distribution of cell proliferation factor expression in different ESD lesions. The positive expression of Ki67 and MCM7 in the evaluated lesions were calculated as percentages; their distributions are displayed herein. Findings in the N/I and LGD groups were statistically significantly different from findings in either HGD or cancers, while there was no difference between N/I and LGD or between HGD and carcinoma in evaluations using the Friedman test (A) (magnification, ×200). Abbreviations: HGD, high-grade dysplasia; LGD, low-grade dysplasia; MCM7, minichromosome maintenance complex component 7; N/I, normal or inflammatory; NS, no statistical significance. The level of statistical significance was indicated as follows: * < 0.05, ** < 0.01, and *** < 0.001.

Proliferation zone expansion with an increasing Ki67 index is generally characteristic of gastric carcinogenesis [20]. In this study, Ki67 and MCM7 expressions were demonstrated using IHC, wherein these factors showed nuclear staining in gastric precancerous and cancerous lesions (Figure 4A). Positive staining for Ki67 and MCM7 was mainly located in the proliferation zone of inflammatory gastric mucosa, whereas scattered nuclear staining was found in LGD. Positive cells were markedly increased in HGD and in cancer cells (Figure 4A). The distributions of Ki67 and MCM7 in each lesion demonstrated that high expression levels were present in HGD and in carcinomas (Figure 4B). Friedman test analysis showed that both N/I and LGD differed from HGD and from carcinomas, while there was no difference between N/I and LGD or between HGD and carcinoma (Figure 4B).

Finally, we found a moderately strong correlation between Ki67 and MCM7 immunostaining (*p* < 0.001, *r* = 0.697, Table 2).

### Expression Profile for SASP, DDR, and Proliferation Factors in Early Gastric Cancers

We performed statistical analyses to clarify correlations between expression levels for SASP, DDR, and proliferation factors in early gastric cancers. The results are summarized in Table 2. cGAS, STING, and IRF3 expression was positively correlated with TP53BP1 expression (*p* = 0.014, *r* = 0.443; *p* = 0.039, *r* = 0.380; and *p* < 0.001, *r* = 0.625), while IRF3 and STAT6 expression was each positively correlated with FANCD2 expression (*p* = 0.008, *r* = 0.477, and *p* = 0.025, *r* = 0.410, respectively). FANCD2 and RPA2 expressions were positively associated with *TP53* mutation status (*p* = 0.005 and *p* = 0.025, respectively) (Figure 3B). Moreover, TP53BP1 and RPA2 expressions was closely correlated with Ki67 (*p* = 0.010, *r* = 0.462; and *p* = 0.041, *r* = 0.375) and MCM7 expression (*p* = 0.011, r = 0.460; and *p* = 0.011, r = 0.458), while FANCD2 expression was positively correlated with Ki67 expression (*p* = 0.024, *r* = 0.412). Finally, IRF3 expression was positively correlated with the expression DDR (TP53BP1, FANCD2, and RPA2) and cell proliferation factors (Ki67 and MCM7) (Table 2).

**FIGURE 5:**
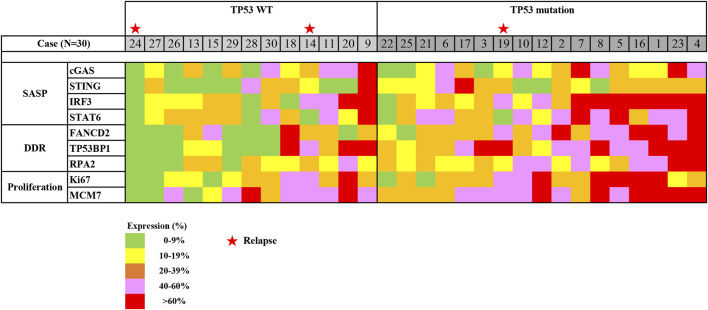
Heatmap of expression profiling in early gastric cancer, using ESD. Expression profiling was generated based on immunostaining for SASP expression, DDR expression, and cell proliferation in regard to TP53 mutation status. Abbreviations: DDR, DNA damage response; ESD, endoscopic submucosal dissection; SASP, senescence-associated secretory phenotype; TP53, tumour protein 53; WT, wild type. Stars indicate cases with relapse (No. 14, 19, and 24).

Next, the expression profile in early gastric cancer was generated based on the expression of SASP, DDR, and proliferation factors in reference to *TP53* mutation status (Figure 5). Roughly, we detected three groups that could be classified as follows: a low expression group with SASP, DDR, and proliferation factors detected at a prevalence of <20%; a moderate expression group with SASP, DDR, and proliferation factors detected at a level ranging from 20% to 60%; and a high expression group with SASP, DDR, and proliferation factors detected in more than 60% of cells. These expression profile groups correlated with *TP53* mutation status, such that low and moderate expression groups were clustered in those with *TP53* wild-type status, while moderate and high expression groups were mainly detected in those positive for *TP53* mutations (Figure 5).

### Analysis of the Associations Between the Expressions of SASP Factors and Clinical Features of Early Gastric Cancer

To identify the possible biological role of the expression of SASP factors in early gastric cancer, associations between the expression of SASP factors and clinical features were estimated in the present study. In following up on the clinical course of each of the 30 cases, we found that four cases underwent subtotal gastrectomy (three cases) or total gastrectomy (one case) following the ESD procedure. Three patients with early gastric cancer relapsed; these patients were determined to have intramucosal carcinoma (pT1a) and well-differentiated adenocarcinoma (without radical operation).

Associations between the expression of SASP factors and other clinicopathological parameters were analysed using either a >10% or >20% cut-off. Cases with lesions located in the gastric pyloric antrum showed a statistically significantly elevated expression of IRF3, as compared with cases with lesions located in the body/fundus (>10%, *p* = 0.024; >20%, *p* = 0.015). Moreover, cases without relapse exhibited a markedly elevated expression of STAT6 (>10%, *p* = 0.014; >20%, *p* = 0.009). Poor differentiated tumour showed a statistically significantly elevated expression of cGAS, as the cut-off value was >10% (*p* = 0.009, detailed data not shown), but not >20% (*p* = 0.142). No statistically significant associations were found between the expression of SASP factors and other clinicopathological parameters, including sex, age, and tumour stage. Results for the >10% threshold are shown in Table 4.

**TABLE 4:** Association between SASP factors and characteristics of patients with early gastric cancer.

	Total *n* = 30	cGAS expression (%)			STING expression (%)			IRF3 expression (%)			STAT6 expression (%)		
		Positive	Negative	*p*-value	Positive	Negative	*p*-value	Positive	Negative	*p*-value	Positive	Negative	*p*-value
		16 (53.3)	14 (46.7)		21 (70.0)	9 (30.0)		20 (66.7)	10 (33.3)		22 (73.3)	8 (26.7)	
Gender													
Male	24 (80.0)	13 (43.3)	11 (36.7)	1	16 (53.3)	8 (26.7)	0.637	17 (56.7)	7 (23.3)	0.372	18 (60.0)	6 (20.0)	0.645
Female	6 (20.0)	3 (10.0)	3 (10.0)		5 (16.7)	1 (3.3)		3 (10.0)	3 (10.0)		4 (13.3)	2 (6.7)	
Age(years)													
≤60	9 (30.0)	5 (16.7)	4 (13.3)	1	6 (20.0)	3 (10.0)	1	6 (20.0)	3 (10.0)	1	8 (26.7)	1 (3.3)	0.374
>60	21 (70.0)	11 (36.7)	10 (33.3)		15 (50.0)	6 (20.0)		14 (46.7)	7 (23.3)		14 (46.7)	7 (23.3)	
Location													
Body/fundus	18 (60.0)	9 (30.0)	9 (30.0)	0.722	11 (36.7)	7 (23.3)	0.249	9 (30.0)	9 (30.0)	**0.024**	14 (46.7)	4 (13.3)	0.678
Antrum/pyloricus	12 (40.0)	7 (23.3)	5 (16.7)		10 (33.3)	2 (6.7)		11 (36.7)	1 (3.3)		8 (26.7)	4 (13.3)	
Differentiation													
Well	19 (63.3)	8 (26.7)	11 (36.7)	0.142	14 (46.7)	5 (16.7)	0.687	12 (40.0)	7 (23.3)	0.702	13 (43.3)	6 (20.0)	0.672
Poor	11 (35.7)	8 (26.7)	3 (10.0)		7 (23.3)	4 (13.3)		8 (26.7)	3 (10.0)		9 (30.0)	2 (6.7)	
Submucosal invasion													
Yes	11 (36.7)	7 (23.3)	4 (13.3)	0.466	7 (23.3)	4 (13.3)	0.687	7 (23.3)	4 (13.3)	1	9 (30.0)	2 (6.7)	0.672
No	19 (63.3)	9 (30.0)	10 (33.3)		14 (46.7)	5 (16.7)		13 (43.3)	6 (20.0)		13 (43.3)	6 (20.0)	
Stage													
pT1a	19 (63.3)	9 (30.0)	10 (33.3)	0.574	14 (46.7)	5 (16.7)	0.708	13 (43.3)	6 (20.0)	0.119	13 (43.3)	6 (20.0)	0.652
pT1b1	7 (23.3)	4 (13.3)	3 (10.0)		4 (13.3)	3 (10.0)		6 (20.0)	1 (3.3)		6 (20.0)	1 (3.3)	
pT1b2	4 (13.3)	3 (10.0)	1 (3.3)		3 (10.0)	1 (3.3)		1 (3.3)	3 (10.0)		3 (10.0)	1 (3.3)	
Relapse													
Yes	3 (10.0)	1 (3.3)	2 (6.7)	0.586	2 (6.7)	1 (3.3)	1	2 (6.7)	1 (3.3)	1	0 (0.0)	3 (10.0)	**0.014**
No	27 (90.0)	15 (50.0)	12 (40.0)		19 (63.3)	8 (26.7)		18 (60.0)	9 (30.0)		22 (73.3)	5 (16.7)	

cGAS, cyclic GMP-AMP synthase; IRF3, interferon-regulatory factor 3; STAT6, signal transducer and activator of transcription 6; STING, cyclic GMP-AMP synthase (cGAS)-stimulator of interferon genes. Cut-off values for positive cases were defined as a prevalence of >10% of positive tumour cells. Statistical evaluations were conducted using Fisher’s test. The bold values indicates statistical significance.

**FIGURE 6:**
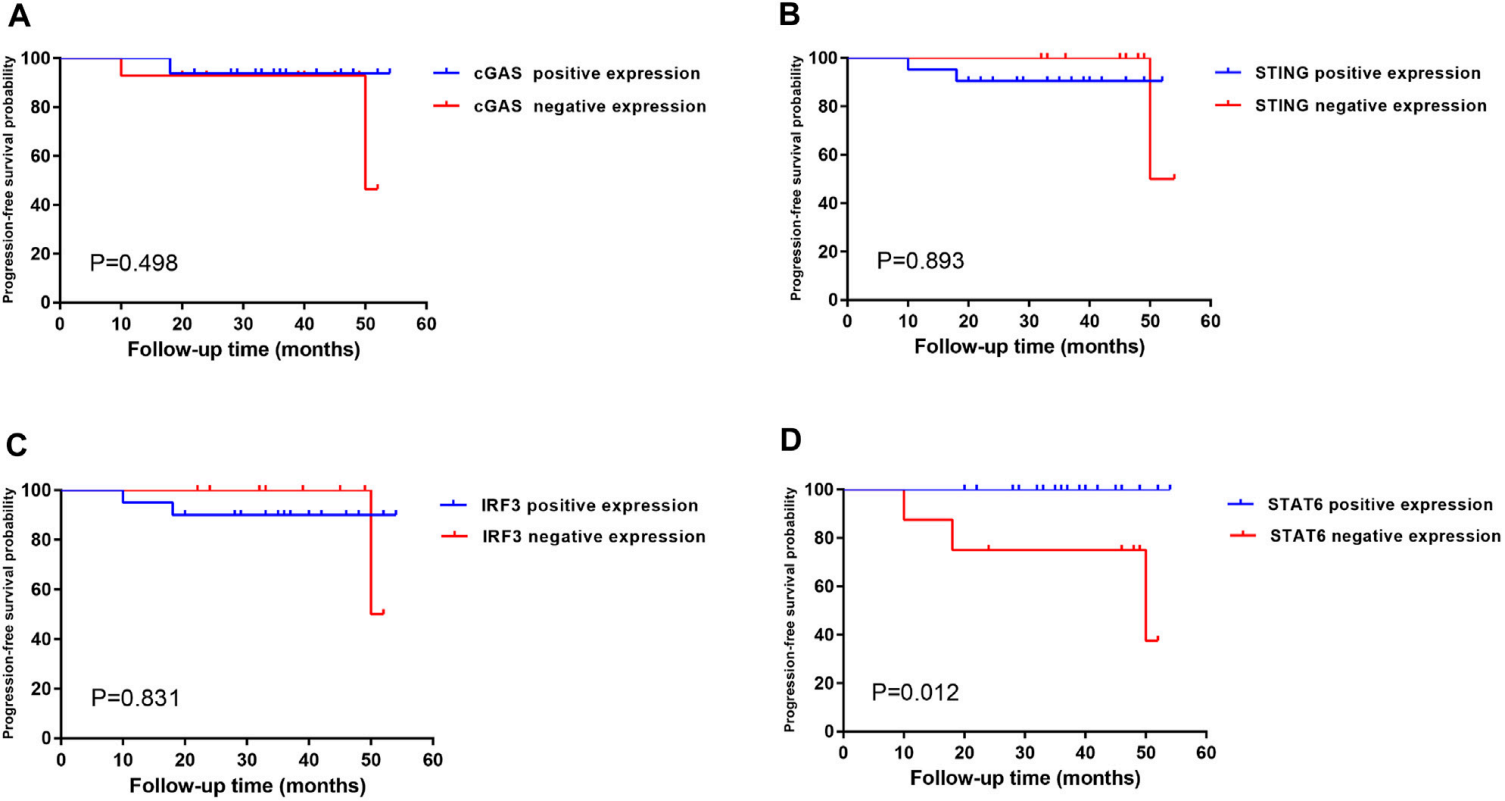
Kaplan–Meier single-factor analysis evaluating progression-free survival in regard to SASP expression in patients with early-stage gastric cancer. Kaplan–Meier single-factor analysis findings were evaluated using log-rank tests. Cut-off values for positive cases were defined as a prevalence of >10% of positive tumour cells. Abbreviations: SASP, senescence-associated secretory phenotype.

Kaplan–Meier single-factor analysis using either a >10% or >20% cut-off and associated log-rank tests revealed that cases showing STAT6-positive expression demonstrated a longer progression-free survival time than those with STAT6-negative expression (>10%, *p* = 0.012; >20%, *p* = 0.025) (results for the >10% threshold are shown in Figure 6), suggesting that STAT6 expression may be an important predictive factor in early gastric carcinoma. cGAS, STING and IRF3 expression showed no statistically significant effect on progression-free survival (>10%, Figure 6). In addition, FANCD2, TP53BP1, RPA2, MCM7, and Ki67 expression as well as *TP53* genomic mutations exhibited no statistically significant effects on progression-free survival (results for the >10% threshold are shown in Supplementary Figure S1).

## Discussion

Gastric cancers are a genetically and phenotypically heterogeneous group of diseases, as revealed in the Cancer Genome Atlas (TCGA) [15] and Asian Cancer Research Group (ACRG) [20] guidelines; these guidelines outline four subtypes of advanced gastric cancers. We note that these subtypes are partially overlapping [15, 21, 22]. However, in clinical practice, it is well known that many gastric cancers develop histologically in a step-wise fashion, progressing from non-neoplastic lesions (such as atrophic gastritis or intestinal metaplasia), to LGD or HGD, to carcinoma *in situ* or invasive lesions. ESD procedures have been developed as effective therapeutic modalities in the evaluation of early gastric cancer. Accumulated ESD specimens also provide an ideal study model, representing the sequential process of gastric carcinogenesis [11, 15, 21, 23].

Previous research has focused on cGAS-STING signalling-mediated SASP activity as a regulation network. Beyond an innate immune reaction, SASP is involved in various pathologic processes, including cancer progression, inflammation, autoimmune disease, and aging. Like other reactive mechanisms, SASP signalling is a double-edged sword in regard to tumorigenesis. More specifically, cellular senescence is considered a barrier against transformation, and the eradication of senescent cells is crucial for the clearance of retaliated tissues [24]. In addition, STING agonists have been demonstrated to suppress cancer growth *in vitro* and *in vivo*, and reactivation of this pathway has been considered a therapeutic approach in cancer treatment [25]. Moreover, there is extensive evidence that SASP promotes the production of pro-inflammatory factors that lead to the remodelling of the tumour microenvironment, thereby promoting tumour progression [5]. Nevertheless, research on the effects of SASP on gastric cancer has focused on advanced cancers, and data from patients with early-stage cancer are still limited.

Using ESD specimens, SASP factors (cGAS, STING, IRF3, and STAT6) were found to be highly expressed, and strong SA-β-gal staining was detected in early gastric cancerous cells as well as in high-grade dysplastic lesions. Elevated expression of cGAS, IRF3, and STAT6 was mainly detected in neoplastic lesions, while STING expression occurred frequently in the surrounding non-neoplastic tissue. Our findings demonstrated the prevalent activity of SASP in early neoplastic lesions, suggesting that SASP activity may mainly be a cancer-related event. We also note that STING staining was high in stromal cells and that careful evaluation is therefore necessary. Antibodies for STING staining in FFPE are currently limited. However, the development of more suitable antibodies is expected.

The signalling cascade for SASP is triggered by cGAS activation, which occurs upon the binding of cGAS to DNA; this cascade has been identified to originate from cytosolic DNA or in the formation of micronuclei due to DNA damage [6]. In reality, DNA damage in gastric epithelial cells is frequently induced by either *H. pylori* infection or chronic inflammation, leading to DDR activation [26, 27]. More importantly, hyper-proliferation of gastric epithelial cells could also induce DNA damage through replication stress, which has been considered the primary source of genome instability and is considered a hallmark of cancer [28, 29]. The stalling of DNA replication forks can generate double-strand breaks and/or single-stranded DNA (ssDNA) gaps, which activate the ataxia-telangiectasia mutated kinase and ataxia telangiectasia and Rad3-related kinase pathways [30, 31]. DNA damage induces the accumulation of many factors, including FANCD2, TP53BP1 and RPA2 as well as phosphorylation of γH2AX and ubiquitylated FANCD2 [32–34]. Thus, DDR plays an essential role in resolving DNA lesions arising from DNA replication stress.

We detected increased expression of FANCD2, TP53BP1, and RPA2 in early gastric cancer, suggesting that DNA damage and DDR are prevalent in the course of this disease. Accelerated cell proliferation and the resultant expansion of the proliferation zone are generally prevalent in early gastric carcinogenesis. Moreover, we proved that the increased expression of MCM7 in early gastric cancer was consistent with the expression of Ki67, reflecting the proliferation status of the evaluated cells. Interestingly, the expression levels of Ki67 and MCM7 statistically correlated with the expression of FANCD2, TP53BP1, and RPA2, indicating that cell proliferation was closely correlated with DNA damage. The expression of TP53BP1 positively correlated with the expression of cGAS, STING, and IRF3, while the expression of FANCD2 positively correlated with the expression of IRF3 and STAT6, suggesting the major role of DDR in SASP activation in early gastric cancers. In addition, SASP activity was positively associated with DDR, suggesting that SASP may be activated by DNA damage in early gastric cancer. The close correlation between DDR and SASP reinforced the observation that SASP could be associated with neoplastic lesions.

*TP53* mutations have been detected in up to nearly two-thirds of evaluated patients with gastric cancer in previous studies, especially in studies conducted in eastern Asia [13, 23]. In our study, 56.67% of the evaluated patients harboured *TP53* gene mutations in known hot spots. HGD and early gastric cancer demonstrated *TP53* gene mutations and p53-positive staining on IHC; this was only detected in HGD and in carcinomas. These findings demonstrate that *TP53* gene mutations occur in the course of the transition between proliferation and dysplasia and in the early stage of gastric carcinogenesis, and are accompanied by the activation of SASP. Furthermore, we conclude that it is reasonable to expect a close correlation between DNA damage and *TP53* mutation status in early gastric carcinogenesis, but not with SASP activity. Cancer cells can develop due to *TP53* gene mutations in the escape of apoptosis or senescence. In this study, *TP53* mutation status was markedly correlated with FANCD2 and RPA2 expression, indicating that replication stress could be involved in the process of acquiring *TP53* mutations. The clear sequential relationship between DDR and *TP53* mutation is still needed to define.

To date, several classification systems for the evaluation of early gastric carcinogenesis have been proposed and implemented clinically; these systems are generally based on morphological changes in gastric epithelia [35]. However, in actual clinical practice, variations have been detected in regard to cell atypia or glandular architecture, especially morphologic differences between intestinal type, foveolar type, or hybrid type presentations [35, 36]. More objective biomarkers, which are yet to be determined, are needed. As revealed in the present study, factors associated with cell proliferation, DDR, SASP, and p53 status differed at the level of statistical significance between LGD and carcinomas, although we detected no differences between HGD and carcinomas. The combination of profound cell proliferation with DDR, SASP, and *TP53* mutations may help in evaluations of the malignant transformation of gastric epithelia.

Consistent distributions of SASP and DDR expression and *TP53* mutations in reference to high levels of proliferation were detected in early gastric cancer cells and HGD, demonstrating the progressive process of gastric carcinogenesis. Detailed analyses regarding these factors in reference to the clinical outcomes of early gastric cancers revealed that cases with STAT6-positive expression showed a longer progression-free survival time than those with STAT6-negative expression. Nevertheless, the progression of cancers is a complicated process, collectively determined by various factors that are interactively fabricated in cancer cells and tumour microenvironments. More interestingly, our recent investigation revealed that cGAS-STING could be activated in severe DNA damage; however, SASP activity has been shown to alternatively mediate DNA autophagy in protecting cancer cell survival [37]. In fact, STAT6 expression is also positively and negatively involved in cancer progression [38].

In summary, our investigation suggests that SASP is prevalent in the neoplastic process of the gastric mucosa, and is stimulated by accelerated cell proliferation-induced DDR. Additionally, we reconfirmed that the combination of profound cell proliferation with DDR and SASP expression and *TP53* mutations (i.e., p53 positivity) could help in defining the malignant transformation of gastric epithelia. Moreover, SASP expression, DNA replication stress, and DDR activity in gastric tumorigenesis may help provide useful insights into the molecular pathogenesis of gastric cancer, providing a preliminary theoretical basis for effecting interventions in early-stage cancer.

## Data Availability

The datasets presented in this study can be found in online repositories. The names of the repository/repositories and accession number(s) can be found in the article/Supplementary Material.

## Supplementary Material

Supplementary Figure S1 Kaplan–Meier single-factor analysis of DDR expression, TP53 mutation status, and cell proliferation in reference to progression-free survival in patients with early gastric cancer. Kaplan–Meier single-factor analysis was carried out using log-rank tests. The cut-off values for positive cases were defined as >10% of positive tumour cells. Abbreviations: DDR, DNA damage response.
