# Supplementary figures and images for: Expression of SASP, DNA Damage Response, and Cell Proliferation Factors in Early Gastric Neoplastic Lesions: Correlations and Clinical Significance
Source: Pathol Oncol Res. 2022 Aug 19;28:1610401. doi: 10.3389/pore.2022.1610401 (PMC9437220; doi:10.3389/pore.2022.1610401)

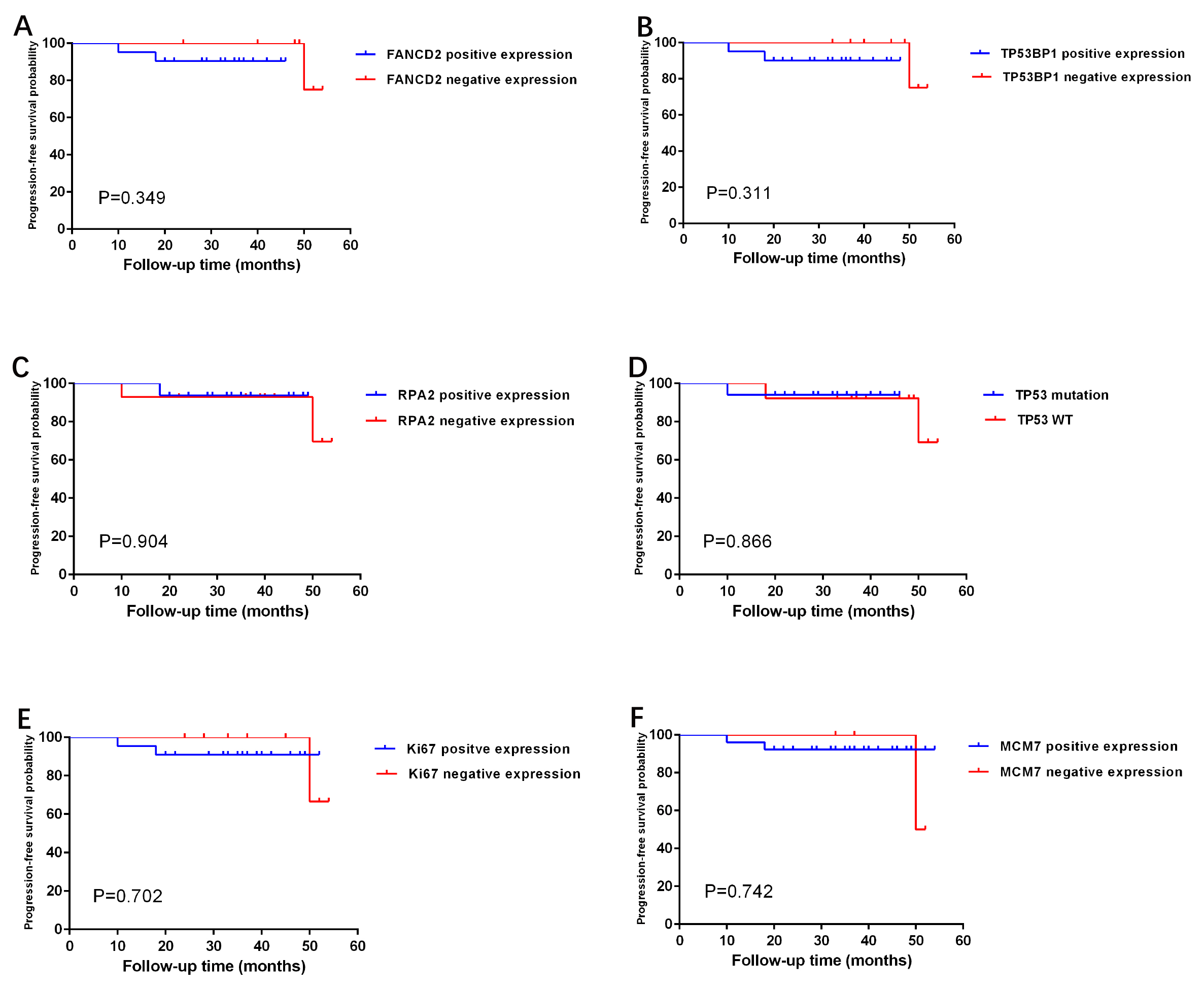

Supplement: Supplementary file 1 [file Image1.TIF]
