# Supplementary material for: Expression of SASP, DNA Damage Response, and Cell Proliferation Factors in Early Gastric Neoplastic Lesions: Correlations and Clinical Significance
Source: Pathol Oncol Res. 2022 Aug 19;28:1610401. doi: 10.3389/pore.2022.1610401 (PMC9437220; doi:10.3389/pore.2022.1610401)
Supplement: Supplementary file 2 [file DataSheet1.docx]

**Supplementary Table 1. Antibodies used in the immunohistochemistry evaluations**

| Primary antibody (Ref.) | Source (No.) | Species | Dilution rate |
| --- | --- | --- | --- |
| cGAS [37] | Santa Cruz Biotechnology (sc-515777) | Mouse monoclonal | 1/500 |
| STING [37] | Abcam (ab181125) | Rabbit monoclonal | 1/1000 |
| IRF3 [37] | Bioworld (BS6921) | Rabbit polyclonal | 1/500 |
| STAT6 [37] | Santa Cruz Biotechnology (sc-374021) | Mouse monoclonal | 1/500 |
| FANCD2 [37] | Abcam (ab108928) | Rabbit monoclonal | 1/100 |
| TP53BP1 [37] | Bioworld (BS90008) | Rabbit polyclonal | 1/200 |
| RPA2 [37] | Sigma-Aldrich (MABE285) | Mouse monoclonal | 1/100 |
| MCM7 [37] | Bioworld (BS6169) | Rabbit polyclonal | 1/200 |
| Ki67 (MIB-1) | Dako (ZM-0166) | Mouse monoclonal | 1/150 |
| P53 (DO7) | Dako (ZM-0408) | Mouse monoclonal | Working solution |

cGAS, cyclic GMP-AMP synthase; FANCD2, Fanconi anemia group D2; IRF3, interferon-regulatory factor 3; MCM7, minichromosome maintenance complex component 7; RPA2, replication protein A; STAT6, signal transducer and activator of transcription 6; STING, cyclic GMP-AMP synthase (cGAS)-stimulator of interferon genes; TP53BP1, tumour suppressor p53 binding protein 1

**Supplementary Table 2. The 41-genes of panel applied in Next generation sequencing**

| TP53 | ATM | AKT1 | BRCA1 | BRCA2 | APC |
| --- | --- | --- | --- | --- | --- |
| KRAS | NRAS | BRAF | PTEN | BLM | BMPR1A |
| CHEK2 | EPCAM | GALNT12 | GREM1 | MLH1 | MSH2 |
| MSH6 | MUTYH | PMS2 | POLD1 | POLE | UGT1A1 |
| SMAD4 | STK11 | CDH1 | EGFR | ERBB2 | HRAS |
| KIT | MET | PDGFRA | PIK3CA | PMS1 | PTCH1 |
| SDHB | SDHC | SCHD | CYP2D6 | DPYD |  |

**Supplementary Table 3. Results of 41-genes mutation of in Next generation sequencing**

| Case No. | TP53 gene mutation | other genes mutation |
| --- | --- | --- |
| 1 | exon5, c.376-2A>G, splicing | WT |
| 2 | exon4, c.159G>A, nonsense | WT |
| 3 | exon6, c.641A>G, missense | APC, exon6,c.637C>T, nonsense; ATM, exon 45, c.6466G>T, nonsense |
| 4 | exon8, Arg273His, missense | WT |
| 5 | exon7, c.733G>A, missense | WT |
| 6 | exon9, c.956del, frame shift | POLE, exon19, c.2041A>T, missense |
| 7 | exon8, c.844C>T, missense | WT |
| 8 | exon8, c.915C>T, nonsense | MET, exon17, c.3508C>T, nonsense |
| 9 | WT | APC, exon16, c.5973del, frame shift |
| 10 | exon7, c.733G>A, missense | WT |
| 11 | WT | WT |
| 12 | exon5, c.491_428del, frame shift | EPCAM, exon3, c.315del, frame shift |
| 13 | WT | WT |
| 14 | WT | WT |
| 15 | WT | BRAC2, exon18, c.8064_8065del, frame shift; ATM, exon30, c.4466G>A, missense; BRAF exon 3, c.358G>A, missense; EGFR, exon28, c.3448A>G, missense; PMS1, exon4 c.316-8del, frame shift; PMS2 exon11, c.1535G>A, missense; POLD1, exon25, c.3115_3117del, frame shift |
| 16 | intron9, c.994-1G>A, splicing | WT |
| 17 | exon8, c.817C>T, missense | WT |
| 18 | WT | PTCH1, exon21, c.3945del, frame shift |
| 19 | exon8，c.844C>T, missense | BRAF, exon18, c.2246C>T, missense |
| 20 | WT | WT |
| 21 | exon5, c.463_466del, frame shift | WT |
| 22 | exon8, c.818G>A, missense | WT |
| 23 | exon8, c.817C>T, missense | PTEN, intron7, c.802-5_802-3del, frame shift |
| 24 | WT | WT |
| 25 | exon7, c.675-5_675dup, frame shift | ERBB2, CN9:1 amplification |
| 26 | WT | WT |
| 27 | WT | ATM, exon15, c.2272_2281del， frame shift; APC, exon17, c.8489G>A, missense; SAMD14-AKT1, rearrangement |
| 28 | WT | KRAS, exon2, c.35G>A, missense; MET, exon2, c.571C>T, missense; MUTYH, exon2, c.128_130del, frame shift |
| 29 | WT | WT |
| 30 | WT | WT |
